# Total Synthesis of Six 3,4-Unsubstituted Coumarins

**DOI:** 10.3390/molecules181215613

**Published:** 2013-12-13

**Authors:** Wenqing Gao, Qingyong Li, Jian Chen, Zhichao Wang, Changlong Hua

**Affiliations:** 1 Key Laboratory of Forest Plant Ecology, Ministry of Education, Northeast Forestry University, Harbin 150040, China; E-Mails: gaowenqing1314@126.com (W.G.); 18042486759@189.com (J.C.); wzc870715@163.com (Z.W.); ghe521@163.com (C.H.); 2 College of Pharmaceutical Science, Zhejiang University of Technology, Hangzhou 310014, China

**Keywords:** 3,4-unsubstituted coumarins, *o*-hydroxybenzaldehyde derivatives, 6,7,8-trimethoxycoumarin, 7-hydroxy-6-ethoxycoumarin

## Abstract

In this article we describe a new methodology for the total synthesis of 3,4-unsubstituted coumarins from commercially available starting materials. Six examples were prepared, including five naturally occurring coumarins—7-hydroxy-6,8-dimethoxy-coumarin (isofraxidin), 7-hydroxy-6-methoxycoumarin (scopoletin), 6,7,8-trimethoxy-coumarin, 6,7-dimethoxycoumarin (scoparone), and 7,8-dihydroxycoumarin (daphnetin) and one synthetic coumarin, 7-hydroxy-6-ethoxycoumarin. Moreover, five important *o*-hydroxybenzaldehyde intermediates were also obtained, namely 2,4-dihydroxy-3,5-dimethoxybenzaldehyde, 2,4-dihydroxy-5-methoxybenzaldehyde, 5-ethoxy-2,4-dihydroxy-benzaldehyde, 2-hydroxy-3,4,5-trimethoxybenzaldehyde, and 2-hydroxy-4,5-dimethoxy-benzaldehyde. The method developed herein involves just three or four steps and allows for the rapid synthesis of these important molecules in excellent yields. This is the first synthesis of 6,7,8-trimethoxycoumarin and 7-hydroxy-6-ethoxycoumarin.

## 1. Introduction

**Figure 1:**
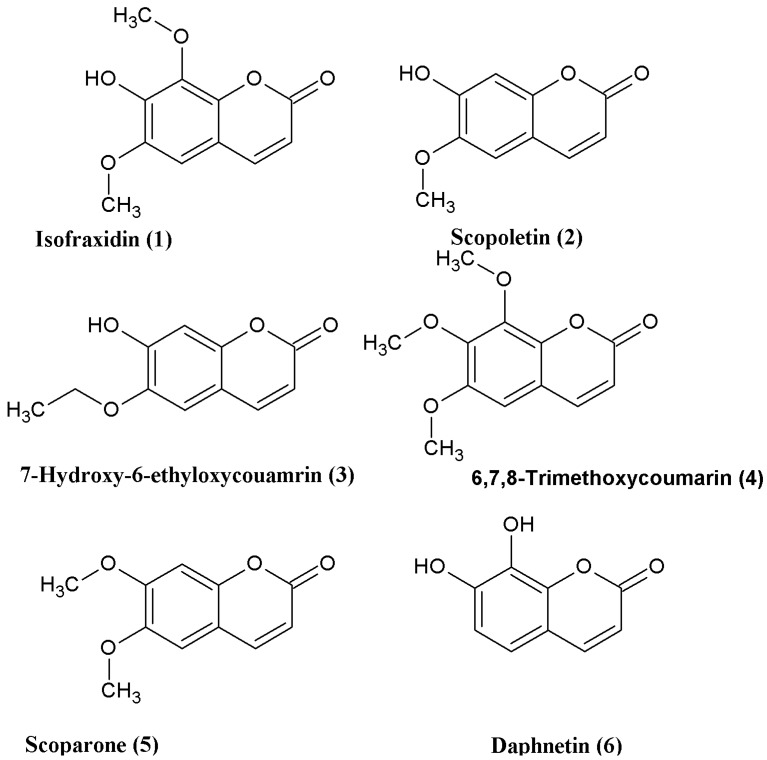
The structure of the synthesized coumarins.

Coumarins (2*H*-1-chromene-2-ones) are abundant in Nature and are common motifs found in drugs, dyes, spices, and agricultural chemicals. In particular, 3,4-unsubstituted coumarins are the most common naturally occurring coumarins, which show potential antimalarial [1], antioxidant [2,3,4,5,6], antimicrobial [7], anti-inflammatory [8], and antitumor [9,10,11] activity, however, their abundance in plants is very low and the purification processes are complex. Therefore, various methods have been developed for the total synthesis of coumarins [11,12,13,14,15,16,17,18,19,20,21,22], including the Perkin [23], Pechmann [24], and Knoevenagel reactions [25]. These reactions mostly lead to coumarins with substituents at the 3- or 4-position. A one-pot Wittig reaction/cyclization has been adopted by de Kimpe for the synthesis of 3,4-unsubstituted coumarins [26]. Herein, we report the highly efficient total synthesis of five naturally occurring coumarins, as well as a synthetic 3,4-unsubstituted coumarin (Figure 1). To the best of our knowledge, this is the first report on the total synthesis of coumarins **3** and **4**.

## 2. Results and Discussion

**Scheme 1:**
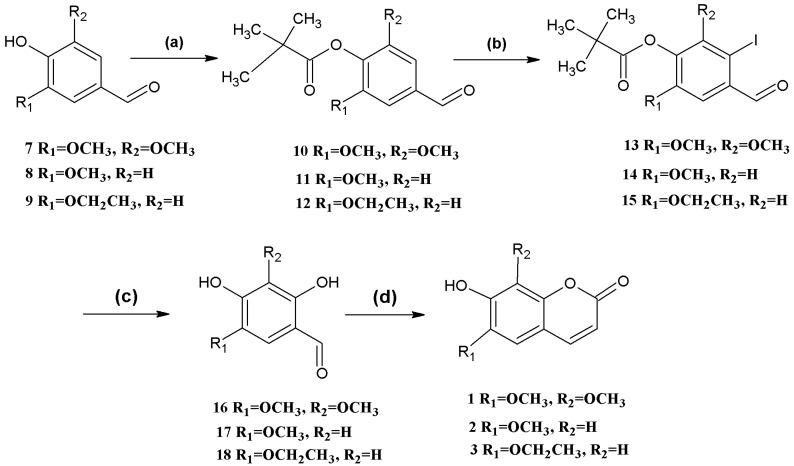
Syntheses of coumarins **1**, **2**, and **3**.

Coumarins **1**, **2**, and **3** were synthesized from simple starting materials (Scheme 1). The hydroxyl group was protected using pivaloyl chloride [27] to give the corresponding products in 100% yield. These compounds were then iodinated in 80%, 77%, 80% yields, respectively, using *N*-iodo-succinimide [28,29], followed by hydrolysis using cuprous oxide, pyridine-2-aldoxime, tetrabutylammonium bromide, and cesium hydroxide [30]. The resulting *o*-hydroxybenzaldehydes were finally reacted with ethyl (triphenylphosphoranylidene) acetate in *N*,*N*-diethylaniline, forming coumarins **1**, **2**, and **3** as described above [26].

*Reagents and conditions*: (a) (CH_3_)_3_COCl, DMAP, Et_3_N, CH_2_Cl_2_; (b) **10** NIS, CF_3_COOH, CH_3_CN, reflux; **11**,**12** *N*-iodosuccinimide (NIS), CF_3_SO_3_H, MeOH; (c) CsOH, *syn*-2-pyridinealdoxime, Cu_2_O, Bu_4_NBr, N_2_, H_2_O; (d) (C_6_H_5_)_3_P=CHCOOCH_2_CH_3_, Et_2_NPh, N_2_, reflux.

**Scheme 2:**
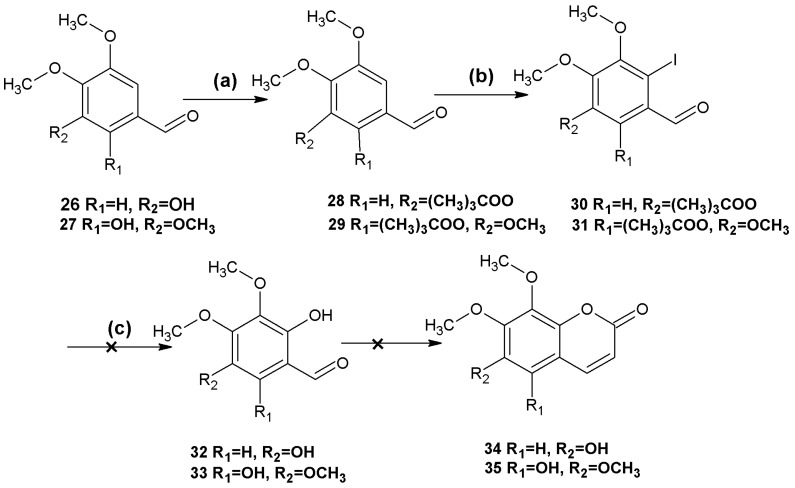
Attempted syntheses of coumarins **34**, **35**.

It was envisaged that this method could be applied to the synthesis of 6,7-dimethoxy-8-hydroxycoumarin (**34**) and 5,6,7,8-tetrahydroxycoumarin (**35**) (Scheme 2).

*Reagents and conditions*: (a) **26** (CH_3_)_3_COCl, DMAP, Et_3_N, CH_2_Cl_2_; **27** MeSO_4_, K_2_CO_3_, CH_3_CN; (b) **28** NIS, CF_3_COOH, CH_3_CN, reflux; **29** NIS, CF_3_SO_3_H, MeOH; (c) CsOH, *syn*-2-pyridinealdoxime, Cu_2_O, Bu_4_NBr, N_2_, H_2_O.

Protection of the hydroxyl groups using pivaloyl chloride and iodination of the resulting compounds proceeded as expected, but hydrolysis of the iodo-compounds **30** and **31** failed to afford the desired products **32** and **33**. It was presumed that both electronic effects and steric hindrance of the three methoxy groups and the hydroxyl group at the *ortho* position of **30** and 3**1** affected the success of these reactions.

**Scheme 3:**
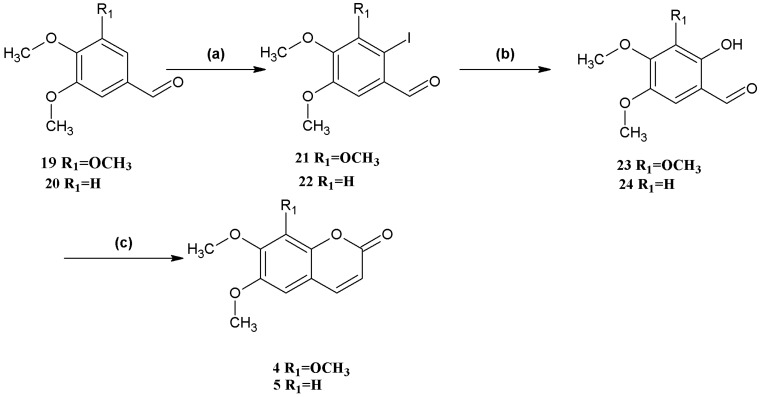
Syntheses of coumarins **4** and **5**.

Synthesis of coumarins **4** and **5** (Scheme 3) began with the iodination of 3,4,5-trimethoxybenzaldehyde (**19**) and veratraldehyde (**20**), respectively, both in 99% yield. Hydrolysis of 2-iodo-3,4,5-trimethoxybenzaldehyde (**21**) and 2-iodo-4,5-dimethoxybenzaldehyde (**22**) afforded 2-hydroxy-3,4,5-trimethoxybenzaldehyde (**23**) and 2-hydroxy-4,5-dimethoxybenzaldehyde (**24**) in 83% and 85% yield, respectively. Finally, the *o*-hydroxybenzaldehydes **23** and **24** were converted to the corresponding coumarins **4** and **5**. The overall yield of **4** and **5** was higher compared to that of compounds **1**–**3**.

*Reagents and conditions*: (a) **19** NIS, CF_3_COOH, CH_3_CN, reflux; **20** NIS, CF_3_SO_3_H, MeOH; (b) CsOH, *syn*-2-pyridinealdoxime, Cu_2_O, Bu_4_NBr, N_2_, H_2_O; (c) (C_6_H_5_)_3_P=CHCOOCH_2_CH_3_, Et_2_NPh, N_2_, reflux.

**Scheme 4:**
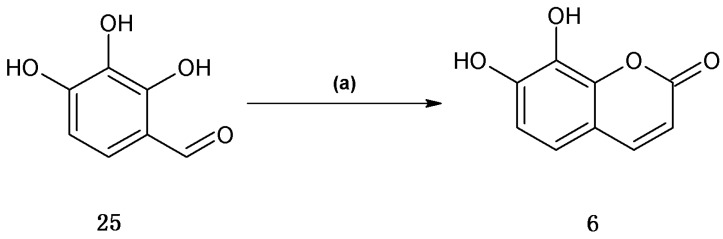
Synthetic procedures of coumarin **6**.

Finally, daphnetin (**6**) was synthesized using the one-pot Wittig/cyclization reaction from commercially available 2,3,4-trihydroxybenzaldhyde in 65% yield (Scheme 4).

*Reagents and conditions*: (a) (C_6_H_5_)_3_P=CHCOOCH2CH3, Et_2_NPh, N_2_, reflux.

This result for daphnetin (**6**) was identical compared to the previously reported synthesis [26]. Similarly for scopoletin (**2**), the overall yield of 50.4% (for four steps) is comparable to that of the previously reported synthesis (52.8%) [24]. However, for isofraxidin (**1**) and scoparone (**5**), the yields of 57.6% and 77.4% are significantly higher compared to those obtained previously [23,25]. The key step in this novel method is the hydrolysis of the iodinated compound, as substituents on the phenyl group have a great influence on this reaction. Activating groups can reduce the probability of hydrolysis and steric hindrance at the *ortho* position also influences the reaction.

## 3. Experimental

All solvents and commercially available reagents were purchased from the suppliers and used without further purification. ^1^H-NMR and ^13^C-NMR spectra were recorded using Bruker DPX 500 (Bruker, Billerica, MA, USA) and 300 spectrometers, respectively. Spectra were recorded in CDCl_3_ and DMSO solutions and chemical shifts are reported in parts per million (ppm) relative to tetramethylsilane (TMS) as the standard. IR spectra were recorded on an Infinity Spectrum One spectrophotometer (Shimadzu, Kyoto, Japan). Mass spectra (Applied Biosystems, Toronto, Canada) were recorded on an Agilent 1100 Series VS (ES, 4000 V) mass spectrometer. Melting points were measured using a Büchi B-450 apparatus (Shanghaishenguang, Shanghai, China). Flash chromatography was performed with ACROS silica gel (particle size 0.030–0.040 mm, pore diameter ca. 6 nm) using a glass column.

### 3.1. General Procedure for the Synthesis of Coumarins **1**–**6**

The appropriate *o*-hydroxybenzaldehyde (1 mmol) and ethyl(triphenylphosphoranylidene) acetate (1.2 mmol) were dissolved in *N*,*N*-diethylaniline (1.5 mL) and the resulting mixture was stirred under a N_2_ atmosphere and reflux for 15 min. The solvent was removed under reduced pressure (1 mmHg, 52 °C) and the resulting brown oil was purified by column chromatography (petroleum ether-ethyl acetate, 3:1).

*7-Hydroxy-6,8-dimethoxy-2H-chromen-2-one* (*Isofraxidin*, **1**)*.* Yellow solid. Yield: 80%. mp (°C): 147–148.5 (lit. 148–149) [13]. ^1^H-NMR (500 MHz, CDCl_3_): 3.95 (s, 3H, OCH_3_), 4.10 (s, 3H, OCH_3_), 6.16 (s, 1H, Ar), 6.29 (d, *J =* 9.5 Hz, 1H, 3-CH), 6.66 (s, 1H, OH), 7.60 (d, *J* = 9.5 Hz, 1H, 4-CH); ^13^C-NMR (75 MHz, CDCl_3_) 56.6 (OCH_3_), 61.2 (OCH_3_), 105.0 (C-5), 110.7 (C-4a), 112.5 (C-3), 135.2 (C-8), 143.5 (C-7), 144.5 (C-8a), 145.3 (C-4), 146.1 (C-6), 160.6 (C-2); IR (KBr, cm^−1^) 1699 (C=O), 1570 (C=C), 3327 (br, OH); HRMS (EI): *m/z* 223 [M+H^+^].

*7-Hydroxy-6-methoxy-2H-chromen-2-one* (*Scopoletin*, **2**)*.* Yellow solid. Yield: 85%. mp (°C): 202.7–204.4 (lit. 204) [15]. ^1^H-NMR (500 MHz, CDCl_3_): 3.82 (s, 3H, OCH_3_), 6.23 (d, *J* = 9.5 Hz, 1H, 3-CH), 6.79 (s, 1H, Ar), 7.23 (s, 1H, Ar), 7.92 (d, *J* = 9.5 Hz, 1H, 4-CH), 10.33 (s, 1H, OH); ^13^C-NMR (75 MHz, CDCl_3_) 56.4 (OCH_3_), 103.2 (C-8), 110.0 (C-5), 111.0 (C-3), 112.1 (C-4a), 144.9 (C-4), 145.7 (C-6), 150.0 (C-7), 151.6 (C-8a), 161.1 (C-2); IR (KBr, cm^−1^) 1558 (C=C), 1683 (C=O), 3334 (br, OH). HRMS (EI): *m/z* 193 [M+H^+^].

*7-Hydroxy-6-ethoxy-2H-chromen-2-one* (**3**)*. *Yellow solid. Yield: 80%. mp (°C): 138.5–142.0. ^1^H-NMR (500 MHz, CDCl_3_): 1.37 (t, *J* = 7.0 Hz, 3H, CH_3_), 4.06 (q, *J* = 7.0 Hz, 2H, OCH_2_-), 6.23 (d, *J* 9.5 Hz, 1H, 3-CH), 6.81 (s, 1H, Ar), 7.90 (d, *J* = 9.5 Hz, 1H, 4-CH), 10.27 (s, 1H, CHO); ^13^C-NMR (75 MHz, CDCl_3_) 15.1 (CH_3_), 64.8 (-CH_2_O-), 103.2 (C-8), 111.0 (C-5), 111.2 (C-3), 112.1 (C-4a), 144.8 (C-4), 144.9 (C-6), 149.9 (C-7), 151.9 (C-8a), 161.1 (C-2); IR (KBr, cm^−1^) 1558 (C=C), 1699 (C=O), 1716, 3383 (br, OH);^.^HRMS (EI): *m/z* 207 [M+H^+^].

*6,7,8-Trimethoxy-2H-chromen-2-one* (*Dimethylfraxetin*, **4**)*.* Yellow solid. Yield: 81%. mp (°C): 103.8–104.5 (lit. 104–105) [31]. ^1^H-NMR (500 MHz, CDCl_3_): 3.91 (s, 3H, OCH_3_), 4.00 (s, 3H, OCH_3_), 4.04 (s, 3H, OCH_3_), 6.35 (d, *J* = 9.5 Hz,1H, 3-CH), 6.69 (s, 1H, Ar), 7.63 (d, *J* = 9.5 Hz, 1H, 4-CH); ^13^C-NMR (75 MHz, CDCl_3_) 56.3 (OCH_3_), 61.5 (OCH_3_), 61.8 (OCH_3_), 103.7 (C-5), 114.3 (C-4a), 115.2 (C-8), 141.2 (C-6), 143.1 (C-7), 143.5 (C-4), 145.9 (C-8), 150.1 (C-8a), 160.5 (C-2); IR (KBr, cm^−1^) 1566 (C=C), 1716 (C=O). HRMS (EI): *m/z* 237 [M+H^+^].

*6,7-Dimethoxy-2H-chromen-2-one* (*Scoparone*, **5**)*.* Yellow solid. Yield: 91%. mp (°C): 142.6–148.4 (lit. 145–146) [18]. ^1^H-NMR (500 MHz, CDCl_3_): 3.81 (s, 3H, OCH_3_), 3.87 (s, 3H, OCH_3_), 6.30 (d, *J* = 9.5 Hz, 1H, 3-CH), 7.07 (s, 1H, Ar), 7.25 (s, 1H, Ar), 7.955 (d, *J* = 9.5 Hz, 1H, 4-CH); ^13^C-NMR (75 MHz, CDCl_3_) 56.3 (OCH_3_), 56.6 (OCH_3_), 100.5 (C-8) 109.3 (C-5), 111.6 (C-4a), 113.1 (C-3), 144.8 (C-6), 146.3 (C-4), 149.9 (C-8a), 153.0 (C-7), 161.0 (C-2); IR (KBr, cm^−1^) 1516 (C=C), 1558, 1708 (C=O), 1616, 3566 (br, OH). HRMS (EI): *m/z* 207 [M+H^+^].

*7,8-Dihydroxy-2H-chromen-2-one* (*Daphnetin*, **6**)*.* Yellow solid. Yield: 65%. mp (°C): 265.7–267.2 (lit. 265−268) [21]. ^1^H-NMR (500 MHz, CDCl_3_): 6.20 (d, *J* = 6.5 Hz, 1H, 3-CH), 6.81 (s, 1H, Ar), 7.03 (s, 1H, Ar), 7.91 (d, *J* = 7.0 Hz, 1H, 4-CH), 9.79 (d, *J =* 7.0 Hz, 1H, OH), 9.80 (d, *J* =7.0Hz, 1H, OH); ^13^C-NMR (75 MHz, CDCl_3_) 111.7 (C-3), 112.5 (C-4a), 112.9 (C-6), 119.3 (C-5), 132.6 (C-8), 144.2 (C-8a), 145.6 (C-4), 150.1 (C-7), 160.9 (C-2); IR (KBr, cm^−1^) 1674 (C=O), 1508 (C=C), 1589. HRMS (EI): *m/z* 179 [M+H^+^].

### 3.2. General Procedure for the Synthesis of Compounds **10**–**12**

The corresponding phenol (3 mmol) was dissolved in dichloromethane (5 mL) and 4-dimethylamiopyridine (0.1 mmol) was added. The reaction was stirred for 0.5 h before addition of pivaloyl chloride (6 mmol) followed by dropwise addition of triethylamine (6 mmol). The reaction mixture was stirred at room temperature for 2 h. The reaction solution was poured into dichloromethane (100 mL) and washed with saturated sodium chloride solution (2 × 100 mL) and saturated sodium bicarbonate solution (2 × 100 mL). The organic phase was collected, dried over anhydrous magnesium sulfate, and filtered, and then the solvent was removed *in vacuo* to obtain compounds **10**–**12**.

*4-Formyl-2,6-dimethoxyphenyl-2,2-dimethylpropanoate* (**10**, C_14_H_18_O_5_)*.* Yellow solid. Yield 100%. mp (°C): 108–110.9. ^1^H-NMR (CDCl_3_, 500 MHz): 1.39 (s, 9H, C(CH_3_)_3_), 3.88 (s, 6H, OCH_3_), 7.14 (s, 2H, Ar), 9.91 (s, 1H, CHO); ^13^C-NMR (75 MHz, CDCl_3_) 27.2 (CH_3_), 29.7 (CH_3_), 39.2 (CC=O), 56.37 (2 × OCH_3_), 106.2 (C-3,5) 134.1 (C-1), 134.4 (C-4), 153.0 (C-2,6), 175.8 (C=O), 191.2 (CHO); IR (KBr, cm^−1^) 1751 (C=O), 1691, 1608; MS (EI): *m/z* 267 [M+H^+^].

*4-Formyl-2-methoxyphenyl-2,2-dimethylpropanoate* (**11**, C_13_H_16_O_4_)*.* White solid. Yield: 100%. mp (°C): 83.8–95.0. ^1^H-NMR (500 MHz, CDCl_3_): 1.38 (s, 9H, C(CH_3_)), 3.88 (s, 3H, OCH_3_), 7.19 (d, *J* = 7.5Hz, 1H, Ar), 7.46 (s, 1H, Ar), 7.48 (d, *J* = 7.5 Hz, 1H, Ar), 9.95 (s, 1H, CHO); ^13^C-NMR (75 MHz, CDCl_3_) 27.2 (3 × CH_3_), 39.2(CC=O), 56.1 (OCH_3_), 110.8 (C-2), 123.4 (C-6), 124.8 (C-5), 135.1 (C-1), 145.6 (C-4), 152.1 (C-3), 176.1 (-COO-), 191.2 (CHO); IR (KBr, cm^−1^) 1689 (C=O), 1749. MS (EI): *m/z* 237 [M+H^+^].

*2-Ethoxy-4-formylphenyl-2,2-dimethylpropanoate* (**12**, C_14_H_18_O_4_)*.* White solid. Yield: 100%. mp (°C): 83.8–85.3. ^1^H-NMR (500 MHz, CDCl_3_): 1.38 (s, 9H, C(CH_3_)_3_), 1.41 (d, *J* = 7.0 Hz, 3H, CH_3_), 4.10 (t, *J* = 7.0 Hz, 2H, OCH_2_-), 7.19 (d, *J* = 8.5 Hz, 1H, Ar), 7.46 (s, 1H, Ar), 7.46 (d, *J =* 7.0 Hz, 1H, Ar), 9.93 (s, 1H, CHO); ^13^C-NMR (75 MHz, CDCl_3_) 14.6 (CH_3_), 27.2 (CH_3_), 39.2 (CC=O), 64.5 (-OCH_2_-), 111.4 (C-2), 123.3 (C-6), 124.6 (C-5), 135.0 (C-1), 145.6 (C-4), 151.4 (C-3), 176.0 (C=O), 191.2 (CHO); IR (KBr, cm^−1^) 1699 (C=O), 1602; MS (EI): *m/z* 251 [M+H^+^].

### 3.3. General Procedure for the Synthesis of **13**, **21**, **22**.

The appropriate benzaldehyde (3 mmol) was dissolved in acetonitrile (5 mL) and *N*-iodosuccinimide (6 mmol) was added. Trifluoroacetic acid (0.3 μmol) was then added dropwise and the reaction mixture was heated under reflux for 6 h. After cooling to room temperature, the reaction solution was quenched by the addition of sodium sulfite (3 mmol) and stirred for five minutes. Subsequently, CH_2_Cl_2_ (100 mL) was added and the solution was extracted with saturated sodium chloride solution (3 × 100 mL). The organic phase was collected, dried over anhydrous magnesium sulfate, and filtered, and the solvent was removed *in vacuo*. Column chromatography (petroleum ether-ethyl acetate, 40:1) afforded the corresponding iodinated compound.

*4-Formyl-3-iodo-2,6-dimethoxyphenyl-2,2-dimethylpropanoate* (**13**, C_14_H_17_O_5_)*.* Yellow solid. Yield: 80%. mp (°C): 100.7–102.5. ^1^H-NMR (500 MHz, CDCl_3_): 1.40 (s, 9H, C(CH_3_)_3_), 3.86 (s, 3H, OCH_3_), 3.85 (s, 3H, OCH_3_), 7.37 (s, 1H, Ar), 10.08 (s, 1H, CHO); ^13^C-NMR (75 MHz, CDCl_3_) 27.1 (CH_3_), 39.3 (CC=O), 56.5 (OCH_3_), 61.5 (OCH_3_), 90.3 (C-2), 108.6 (C-6), 133.0 (C-4), 139.0 (C-1), 153.0 (C-5), 152.3 (C-3), 175.3 (C=O), 191.0 (C=O), 195.2 (C=O); IR (KBr, cm^−1^) 1691 (C=O), 1759; MS (EI): *m/z* 393 [M+H^+^].

*2-Iodo-3,4,5-trimethoxybenzaldehyde* (**21**, C_10_H_11_O_3_)*.* Yellow solid. Yield: 99%. mp (°C): 67.6–68.1. ^1^H-NMR (500 MHz, CDCl_3_): 3.97 (s, 3H, OCH_3_), 3.92 (s, 3H, OCH_3_), 3.91 (s, 3H, OCH_3_), 7.35 (s, 1H, OCH_3_), 10.05 (s, 1H, CHO); ^13^C-NMR (75 MHz, CDCl_3_) 61.2 (OCH_3_), 61.0 (OCH_3_), 56.3 (OCH_3_), 91.6 (C-2), 108.6 (C-6), 130.6 (C-1), 147.8 (C-4), 153.0 (C-5), 154.1 (C-3), 195.3 (CHO); IR (KBr, cm^−1^) 1687 (C=O); MS (EI): *m/z* 323 [M+H^+^].

*2-Iodo-4,5-dimethoxybenzaldehyde* (**22**, C_9_H_9_O_2_)*.* Yellow solid. Yield: 99%. mp (°C):: 145.1–145.5. ^1^H-NMR (500 MHz, CDCl_3_): 3.92 (s, 3H, OCH_3_), 3.96 (s, 3H, OCH_3_), 7.31 (s, 1H, Ar), 7.42 (s, 1H, Ar), 9.87 (s, 1H, Ar); ^13^C-NMR (75 MHz, CDCl_3_) 56.1 (OCH_3_), 56.5 (OCH_3_), 92.8 (C-4), 111.1 (C-5), 121.8 (C-1), 128.4 (C-6), 149.8 (C-3), 154.5 (C-2), 194.9 (CHO); IR (KBr, cm^−1^) 1672 (C=O); MS (EI): *m/z* 293 [M+H^+^].

### 3.4. General Procedure for the Synthesis of **14** and **15**

The appropriate benzaldehyde (3 mmol) was dissolved in methanol (20 mL) and *N*-iodosuccinimide (6 mmol) was added. Trifluoromethanesulfonic acid (6 mmol) was then added dropwise and the reaction mixture was stirred at room temperature for 6 h. The reaction was quenched by the addition of sodium sulfite and the reaction mixture was stirred for five minutes. Subsequently, dichloromethane (100 mL) was added and the solution was extracted with saturated sodium chloride solution (3 × 100 mL). The organic phase was collected, dried over anhydrous magnesium sulfate, and filtered; then, the solvent was removed *in vacuo*. Column chromatography (petroleum ether-ethyl acetate, 40:1) afforded the corresponding iodinated compound.

*4-Formyl-5-iodo-2-methoxyphenyl-2,2-dimethylpropanoate* (**14**, C_13_H_15_O_4_)*.* Yellow solid. Yield: 77%. mp (°C): 98.4. ^1^H-NMR (500 MHz, CDCl_3_): 1.36 (s, 9H, C(CH_3_)_3_), 3.86 (s, 3H, OCH_3_), 7.50 (s, 1H, Ar), 7.58 (s, 1H, Ar), 9.96 (s, 1H, CHO); ^13^C-NMR (75 MHz, CDCl_3_) 27.1 (3 × CH_3_), 39.2 (CC=O), 56.2 (OCH_3_), 89.6 (C-2), 112.7 (C-6), 133.2 (C-3), 134.1 (C-1), 145.7 (C-4), 152.3 (C-5), 175.7 (-COO-), 194.9 (CHO); IR (KBr, cm^−1^) 1693 (C=O), 1751; MS (EI): *m/z* 363 [M+H^+^].

*2-Ethoxy-4-formyl-5-iodophenyl-2,2-dimethylpropanoate* (**15**, C_14_H_17_O_4_)*.* Yellow solid. Yield: 80%. mp (°C): 81.7–83.8. ^1^H-NMR (500 MHz, CDCl_3_): 1.37 (s, 9H, C(CH_3_)_3_), 1.39 (t, *J =* 7.0 Hz, 3H, -CH_3_), 4.08 (q, *J* = 7.0 Hz, 2H, OCH_2_-), 7.47 (s, 1H, Ar), 7.58 (s, 1H, Ar), 9.95 (s, 1H, CHO); ^13^C-NMR (75 MHz, CDCl_3_) 14.5 (CH_3_), 27.1 (CH_3_), 39.2 (CC=O), 64.7 (-OCH_2_-), 89.4 (C-2), 113.3 (C-6), 133.1 (C-3), 134.0 (C-1), 145.8 (C-4), 151.6 (C-5), 175.7 (C=O), 195.0 (CHO); IR (KBr, cm^−1^) 1685 (C=O), 1759; MS (EI): *m/z* 377 [M+H^+^].

### 3.5. General Procedure for the Synthesis of **16**–**18**, **23** and **24**

To the appropriate iodinated compound (2 mmol) was added Cu_2_O (0.1 mmol), pyridine-2-aldoxime (0.2 mmol), tetrabutylammonium bromide (0.4 mmol), cesium hydroxide monohydrate (10 mmol), and water (2 mL) and the mixture was stirred under N_2_ atmosphere for 10 h. The reaction mixture was acidified to pH 5–7 with 1 M hydrochloric acid. The aqueous layer was extracted with dichloromethane (3 × 100 mL). The organic phases were combined, dried over anhydrous magnesium sulfate, and filtered; the solvent was then removed *in vacuo*. Column chromatography (petroleum ether-ethyl acetate: 20:1) afforded the corresponding phenol.

*2,4-Dihydroxy-3,5-dimethoxybenzaldehyde* (**16**, C_9_H_10_O_5_)*.* Yellow solid. Yield: 90%. mp (°C): 87.5–89.8. ^1^H-NMR (500 MHz, CDCl_3_): 3.91 (s, 3H, OCH_3_), 4.01 (s, 3H, OCH_3_), 6.41 (s, 1H, Ar), 6.75 (s, 1H, OH), 9.70 (s, 1H, OH), 11.30 (s, 1H, CHO); ^13^C-NMR (75 MHz, CDCl_3_) 56.6 (OCH_3_), 60.9 (OCH_3_), 108.9 (C-6), 112.9 (C-1), 134.6 (C-3), 141.2 (C-4), 146.9 (C-2), 151.6 (C-5), 194.6 (CHO); IR (KBr, cm^−1^) 3350 (br, OH), 1650 (C=O); MS (EI): *m/z* 199 [M+H^+^].

*2,4-Dihydroxy-5-methoxybenzaldehyde* (**17**, C_8_H_8_O_4_)*.* Yellow solid. Yield: 77%. mp (°C): 152.1−153.2. ^1^H-NMR (500 MHz, CDCl_3_): 3.92 (s, 3H, OCH_3_), 6.41 (s, 1H, Ar), 6.53 (s, 1H, Ar), 6.89 (s, 1H, OH), 9.68 (s, 1H, OH), 11.34 (s, 1H, CHO); ^13^C-NMR (75 MHz, CDCl_3_) 56.5 (OCH_3_), 103.2 (C-3), 112.9 (C-1), 113.3 (C-6), 140.7 (C-5), 154.4 (C-4), 159.6 (C-2), 193.8 (CHO); IR (KBr, cm^−1^) 3292 (br, OH), 1635 (C=O); MS (EI): *m/z* 169 [M+H^+^].

*5-Ethoxy-2,4-dihydroxybenzaldehyde* (**18**, C_9_H_10_O_4_)*.* Yellow solid. Yield: 77%. mp (°C): 131.8−134.4. ^1^H-NMR (500 MHz, CDCl_3_): 1.47 (t, *J* = 7.0 Hz, 3H, CH_3_), 4.12 (q, *J* = 7.0 Hz, 2H, OCH_2_-), 6.49 (s, 1H, Ar), 6.53 (s, 1H, Ar), 6.88 (s, 1H, OH), 9.65 (s, 1H, OH), 11.33 (s, 1H, CHO); ^13^C-NMR (75 MHz, CDCl_3_) 14.8 (CH_3_), 65.3 (-CH_2_O), 103.1 (C-3), 113.3 (C-1), 113.9 (C-6), 139.9 (C-5), 154.6 (C-4), 159.5 (C-2), 193.8 (CHO); IR (KBr, cm^−1^) 1627 (C=O), 1699, 3367 (br, OH); MS (EI): *m/z* 183 [M+H^+^].

*2-Hydroxy-3,4,5-trimethoxybenzaldehyde* (**23**, C_10_H_12_O_4_)*.* Yellow solid. Yield: 83%. mp (°C): 46.0–46.6. ^1^H-NMR (500 MHz, CDCl_3_): 3.87 (s, 3H, OCH_3_), 3.94 (s, 3H, OCH_3_), 4.05 (s, 3H, OCH_3_), 6.78 (s, 1H), 9.78 (s, 1H, OH), 11.01 (s, 1H, CHO); ^13^C-NMR (75 MHz, CDCl_3_) 61.4 (OCH_3_), 61.1 (OCH_3_), 56.5 (OCH_3_), 109.2 (C-6), 115.2 (C-1), 141.0 (C-3), 146.3 (C-4), 150.3 (C-2), 151.8 (C-5), 194.9 (CHO); IR (KBr, cm^−1^) 1641 (C=O), 3240 (br, OH); MS (EI): *m/z* 213 [M+H^+^].

*2-Hydroxy-4,5-dimethoxybenzaldehyde* (**24**, C_9_H_10_O_3_)*.* Yellow solid. Yield: 85%. mp (°C): 105.5–112.8. ^1^H-NMR (500 MHz, CDCl_3_): 3.88 (s, 3H, OCH_3_), 3.94 (s, 3H, OCH_3_), 6.48 (s, 1H, Ar), 6.91 (s, 1H, Ar), 9.71 (s, 1H, OH), 11.40 (s, 1H, CHO); ^13^C-NMR (75 MHz, CDCl_3_) 56.4 (OCH_3_), 56.4 (OCH_3_), 100.1 (C-3), 112.9 (C-1), 113.2 (C-6), 143.0 (C-5), 157.2 (C-2), 159.4 (C-4), 194.0 (CHO); IR (KBr, cm^−1^) 1624 (C=O), 3446 (br, OH); MS (EI): *m/z* 183 [M+H^+^].

## 4. Conclusions

A novel method for the total synthesis of six coumarins and five important intermediates has been developed. In most cases, the overall yields of these products are higher than those previously reported. Furthermore, this is the first reported total synthesis of 7-hydroxy-6-ethoxycoumarin (**3**) and 6,7,8-trimethoxycoumarin (**4**). All of these coumarins and advanced intermediates will be evaluated for their activity, and the results will be reported in due course.
